# Rethinking running biomechanics: a critical review of ground reaction forces, tibial bone loading, and the role of wearable sensors

**DOI:** 10.3389/fbioe.2024.1377383

**Published:** 2024-04-08

**Authors:** Liangliang Xiang, Zixiang Gao, Alan Wang, Vickie Shim, Gusztáv Fekete, Yaodong Gu, Justin Fernandez

**Affiliations:** ^1^ Department of Radiology, Ningbo No. 2 Hospital, Ningbo, China; ^2^ Auckland Bioengineering Institute, The University of Auckland, Auckland, New Zealand; ^3^ Faculty of Engineering, University of Pannonia, Veszprém, Hungary; ^4^ Center for Medical Imaging, Faculty of Medical and Health Sciences, The University of Auckland, Auckland, New Zealand; ^5^ Vehicle Industry Research Center, Széchenyi István University, Győr, Hungary; ^6^ Faculty of Sports Science, Ningbo University, Ningbo, China; ^7^ Department of Engineering Science, The University of Auckland, Auckland, New Zealand

**Keywords:** impact load, tibial acceleration, inertial measurement unit (IMU) sensor, machine learning, running

## Abstract

This study presents a comprehensive review of the correlation between tibial acceleration (TA), ground reaction forces (GRF), and tibial bone loading, emphasizing the critical role of wearable sensor technology in accurately measuring these biomechanical forces in the context of running. This systematic review and meta-analysis searched various electronic databases (PubMed, SPORTDiscus, Scopus, IEEE Xplore, and ScienceDirect) to identify relevant studies. It critically evaluates existing research on GRF and tibial acceleration (TA) as indicators of running-related injuries, revealing mixed findings. Intriguingly, recent empirical data indicate only a marginal link between GRF, TA, and tibial bone stress, thus challenging the conventional understanding in this field. The study also highlights the limitations of current biomechanical models and methodologies, proposing a paradigm shift towards more holistic and integrated approaches. The study underscores wearable sensors’ potential, enhanced by machine learning, in transforming the monitoring, prevention, and rehabilitation of running-related injuries.

## 1 Introduction

The external loading generated during locomotion is essential for generating momentum necessary for movements such as propelling, braking, and changing direction. Metrics of ground reaction forces (GRF) are crucial in understanding the biomechanical mechanisms during running (Johnson C. D. et al., 2020). This understanding plays a pivotal role in preventing musculoskeletal injuries and in evaluating rehabilitation processes (Van der Worp et al., 2016; Willwacher et al., 2022; Pan et al., 2023; Yang et al., 2023). Proper analysis and interpretation of these reaction forces can provide invaluable insights into the efficiency and safety of movement, thus informing strategies for injury prevention and the effectiveness of rehabilitation techniques (Zadpoor and Nikooyan, 2011; Johnson C. D. et al., 2020).

The piezoelectric force plate is a widely recognized and direct method for assessing external loading in biomechanical contexts (Novacheck, 1998). This technology operates on the principle that an applied force results in sensor distortion on the plate, leading to measurable voltage changes proportional to the force’s intensity (Bobbert and Schamhardt, 1990). These force plates are instrumental in capturing three-dimensional force and moment data, which are essential for conducting inverse dynamics analyses (Delp et al., 2007). Inverse dynamics is a standard process in motion analysis where the net moment at body joints is calculated based on their acceleration and velocity. This approach is crucial for understanding the mechanics of movement and the forces acting upon the body’s joints (Delp et al., 2007). In addition, the assessment of static loads is also considered a non-negligible issue in postural control rehabilitation and athletic training. A previous study (Martelli et al., 2011) underscores the critical influence of sub-optimal neuromotor control strategies on the internal load dynamics of the hip joint during regular walking activities, suggesting a potential for significantly elevated fracture risks beyond what is estimable through external loading measurements alone.

Gait lab-based kinetic measurements have been used as indictors to assess tibial acceleration (TA), which is utilized for quantifying shock attenuation (Hennig and Lafortune, 1991; Lafortune et al., 1995; Xiang et al., 2022c). The impact shock has been discussed linked with the incidence of chronic overuse injuries (Hennig et al., 1993). Given the advances of wearable technology in the past twenty decades, trial-axis acceleration and angular velocity could be measured from accelerometer and gyroscope in a single inertial sensor (Afaq et al., 2020; Xiang et al., 2022d; Xiang et al., 2022e; Mason et al., 2023; Xiang et al., 2024; Yamane et al., 2024). This made segment acceleration measurements easier and more convenient, shifting the question to: Can we use portable and affordable inertial sensors to evaluate external loading rather than the force plate, which is conventionally embedded in the floor in a gait lab and is cost-prohibitive (Sheerin et al., 2019; Hutabarat et al., 2021; Xiang et al., 2022e)?

Many studies have been conducted attempting to address this question. Johnson et al. (2023) demonstrated a moderate correlation between vertical loading rates and peak vertical TA during walking with load carriage. Tenforde et al. (2020) found that vertical TA could seers as a reliable indicator of load rates in runners with injuries, regardless of their varying foot strike patterns, based on the correlation of coefficient. The findings from Johnson et al. (2021) showed a strong correlation between TA and instantaneous loading rates in the medal-lateral axis while running on a treadmill with rearfoot strike style. Van den Berghe et al. (2019) demonstrated axial and resultant peak TA are highly correlated to peak vertical impact loading rate across different overground running speeds.

Contrarily, recent empirical studies, such as the one by Zandbergen et al. (2023), show no correlation between peak TA and tibial compressive forces. Similarly, Matijevich et al. (2019) demonstrated that metrics of GRF are not strongly correlated with tibial bone load. Therefore, linking GRF metrics with tibial forces or the risk of overuse injuries during running may be misleading (Matijevich et al., 2019).

**FIGURE 1:**
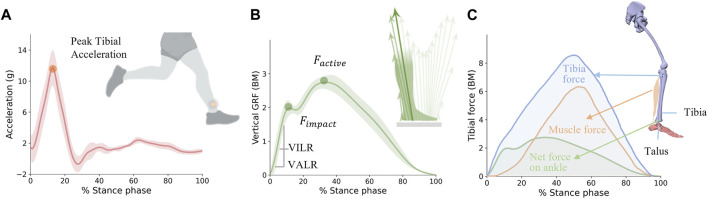
An illustration depicting **(A)** vertical tibial acceleration, **(B)** vertical ground reaction force, and **(C)** tibial force during running.

This leads to a paradox: if TA is an index of running injuries, associated with impact loading rate, then why is there no correlation between TA and tibial bone loading, which is a crucial parameter for tibial stress fractures during running? In other words, while external acceleration is associated with generated external force, it does not correlate with internal force on tibial bone loading (Matijevich et al., 2019; Sheerin et al., 2019; Zandbergen et al., 2023). Therefore, the biomechanics or sports medicine community may need to reconsider whether external acceleration should be an indicator for running injuries, or if internal adaptation is more significant in causing injuries (Matijevich et al., 2019) (Figure 1).

One of the most significant advancements in biomechanics facilitated by wearable sensors is their capability to enable data-driven approaches, offering portable and innovative solution (Halilaj et al., 2018; Gholami et al., 2020; Hernandez et al., 2021; Xiang et al., 2022e; Mason et al., 2023; Xiang et al., 2023). Notably, the prediction of GRF metrics from inertial sensors using deep learning algorithms has shown high accuracy, as evidenced in studies (Ngoh et al., 2018; Johnson W. R. et al., 2020; Tan et al., 2020). Similarly, projections of inner tibial bone load have been successfully explored through machine learning (Matijevich et al., 2020). Understanding the role of external TA in both external impact loading and internal tibial bone loading, therefore, becomes crucial (Matijevich et al., 2019). Enhancing the evaluation of these factors through machine learning not only presents an intriguing area of research but also holds substantial potential implications for future applications in sports medicine, injury prevention, and rehabilitation strategies (Zadpoor and Nikooyan, 2011; Johnson C. D. et al., 2020; Xiang et al., 2022a; Xiang et al., 2022b; Gao et al., 2023; Lloyd et al., 2023; Uhlrich et al., 2023; Xiang et al., 2023).

This systematic review aims to bridge a critical gap in our understanding of the relationship among GRF, TA, tibial bone loading, and running-related injuries, a topic of significant importance to both athletes and recreational runners. By focusing on the burgeoning role of wearable technology in this domain, we seek to analyze and synthesize recent advancements in this field, considering their increased accessibility and application in both research and practical settings. Our review will methodically examine existing literature, employing rigorous criteria to evaluate the validity and reliability of various measurement techniques. Ultimately, this review endeavors to provide valuable insights into running mechanics and injury prevention, potentially informing future research directions, training methodologies, and rehabilitative practices, thereby leveraging the latest advancements in technology and data analysis.

## 2 Methods

The protocol of this systematic review was designed in alignment with the Preferred Reporting Items for Systematic Reviews and Meta-Analysis (PRISMA) guidelines (Moher et al., 2010). Additionally, the protocol was officially registered with PROSPERO (Registration Number: CRD42023483210).

### 2.1 Search strategy

PubMed, Scopus, SPORTDiscus, and IEEE Xplore electronic databases were searched for the period from 2000 to November 2023, using the specified terms combined with the Boolean operators outlined in Table 1. Additionally, relevant studies were identified by reviewing bibliographies in academic articles. The titles, abstracts, and full texts of the retrieved documents were meticulously evaluated to determine their relevance. Only papers published in English that specifically measured TA/tibial loading and GRF in the context of running were considered. Exclusion criteria included papers that exclusively assessed GRF signals, those with sensor placements other than the tibial region, and studies involving participants using any form of aid or equipment during running.

**TABLE 1:** Electronic databases retrieve strategy.

Search items	Limit conditions
**PubM** **ed, Scopus, SPORTDiscus and IEEE Xplore** (“wearable sensor” OR “inertial sensor” OR “accelerometer” OR “acceleration” OR “IMU”) AND (“tibia*” OR “tibial load*” OR “tibial force*” OR “tibial bone load*” OR “tibial bone force*” OR “tibial compression force”) AND (“ground reaction force*” OR “reaction force*” OR “external load*” OR “GRF” OR “loading rate” OR “impact loading” OR “impact peak” OR “active peak” OR “braking force” OR “propulsive force”) AND (“running” OR “runner*” OR “jog” OR “jogging”)	Keywords in all field of the article; Advanced search; Article type: Journal; Language: English; Publish time: From 2000 to November 2023
**ScienceDirect** (“wearable sensor” OR “inertial sensor” OR “accelerometer” OR “IMU”) AND (“tibia” or “Tibial”) and “reaction force” OR “GRF”) and (“running” OR “runner” OR “jogging”)	Keywords in all field of the article; Advanced search; Article type: Journal; Language: English; Publish time: From 2000 to November 2023

Bold values are electronic databases.

### 2.2 Eligibility criteria

In accordance with the Participants, Intervention, Comparisons, and Outcomes (PICO) criteria, information was extracted from thirteen included studies. This extraction focused on participant details, correlation variables, and data-driven approaches. The participant information encompassed the number of participants, their gender, age, height, weight, and running speed during data collection. The Pearson correlation coefficient was used for the correlation evaluation in included studies. The correlation variable included data calculated by the acceleration sensor and/or the force plate, as well as running conditions (speeds and surfaces) for data collection. Machine learning including deep learning were extracted from the included studies. The calculation of the Vertical Average Loading Rate (VALR) is based on the gradient of the initial impact transient, specifically over its linear section, typically spanning from 20% to 80% of the vertical impact peak. In contrast, the Vertical Instantaneous Loading Rate (VILR) is determined by identifying the maximum slope between any two consecutive data points within the same region of interest (Davis et al., 2015).

Two independent reviewers (Z.G. and L.X.) conducted the selection process. Disagreements between these authors regarding article inclusion were resolved through further discussion. In cases where consensus was unattainable, a third reviewer (J.F.) was consulted for resolution. Studies were excluded if they met the following criteria: 1) Participants exhibiting physical injuries during testing; 2) TA measured from the proximal tibia or medial aspect of the distal tibia; 3) Absence of correlation or data-driven approaches; 4) Studies that scored below 75% in the quality assessment. The collation of articles and the removal of duplicates were carried out using EndNote X9 (Thomson Reuters, Carlsbad, California, United States).

### 2.3 Quality assessment

The assessment protocol for appraising the quality of the included articles was based on a modified version of established scales in the fields of sports science, healthcare, and rehabilitation. This approach, commonly used in analyzing studies in an exercise-based training context, adopted the study quality scoring system developed by Black et al. (2016). Two assessors, Z.G. and L.X., independently employed this scoring system to evaluate the quality of the graded articles. The results were then reviewed and confirmed by a third reviewer (J.F.). The evaluation included nine distinct criteria, each contributing to a cumulative score (range: 0–18). The criteria were as follows: (1) inclusion criteria stated (score: 0–2); (2) appropriate assignment of subjects (random/equal baseline); (3) description of intervention; (4) definition of dependent variables; (5) practicality of assessments; (6) practicality of training duration (acute vs. long term); (7) appropriateness of statistics (variability, repeated measures); (8) detailed results (mean, standard deviation, percent change, effect size); (9) insightful conclusions (clear, concise, future directions), with each criterion graded from 0 (no) to 1 (maybe) or 2 (yes). To maintain impartiality in the quality assessment of the included studies, the scores were converted to a percentage scale, ranging from 0% to 100%.

### 2.4 Data synthesis

#### 2.4.1 Data processing and subgroup analysis

Fisher’s Z transformation is utilized in meta-analysis to synthesize correlation coefficients from diverse studies. This transformation stabilizes the variance of the correlation coefficients, effectively converting them to a scale where they approximate a normal distribution. Consequently, this method facilitates a more precise and dependable estimation of the overall correlation across the compiled studies. In meta-analysis, moderator analysis was performed to analyze the factors of running surface (overground and treadmill running) and foot strike patterns (RFS: rearfoot strike pattern, MFS: midfoot strike pattern, and FFS: forefoot strike pattern). That might influence the size or direction of the effect between vertical peak TA and GRF, i.e., VALR and VILR.

The I^2^ statistic quantifies the percentage of total variation across studies attributable to heterogeneity rather than random chance. Conventionally, I^2^ values of 25%, 50%, and 75% are interpreted as indicative of low, moderate, and high heterogeneity, respectively (Higgins et al., 2003). Tau-squared (τ^2^) serves as an estimate of the variance between studies within the framework of a random-effects model, with larger τ^2^ values signaling increased heterogeneity. For all tests conducted, an alpha level of 0.05 was established to determine statistical significance. The meta-analysis was conducted using the Meta statistical analysis package in R (version 4.3.2, R Foundation for Statistical Computing, Vienna, Austria).

#### 2.4.2 Sensitivity analysis

Sensitivity analyses were performed to identify potential factors contributing to the observed high heterogeneity and to assess the robustness of the synthesized results. This involved conducting the analysis multiple times, each time sequentially excluding the study with the lowest weight, and then the two studies with the lowest weights, and so on, until the n-1 studies with the lowest weights were excluded (where n equals the total number of included studies). Considering the diversity in the studies included in this review and the variation in effect sizes from one study to another, random effects models were employed in the meta-analysis to accommodate these discrepancies.

## 3 Results

### 3.1 Search results

**FIGURE 2:**
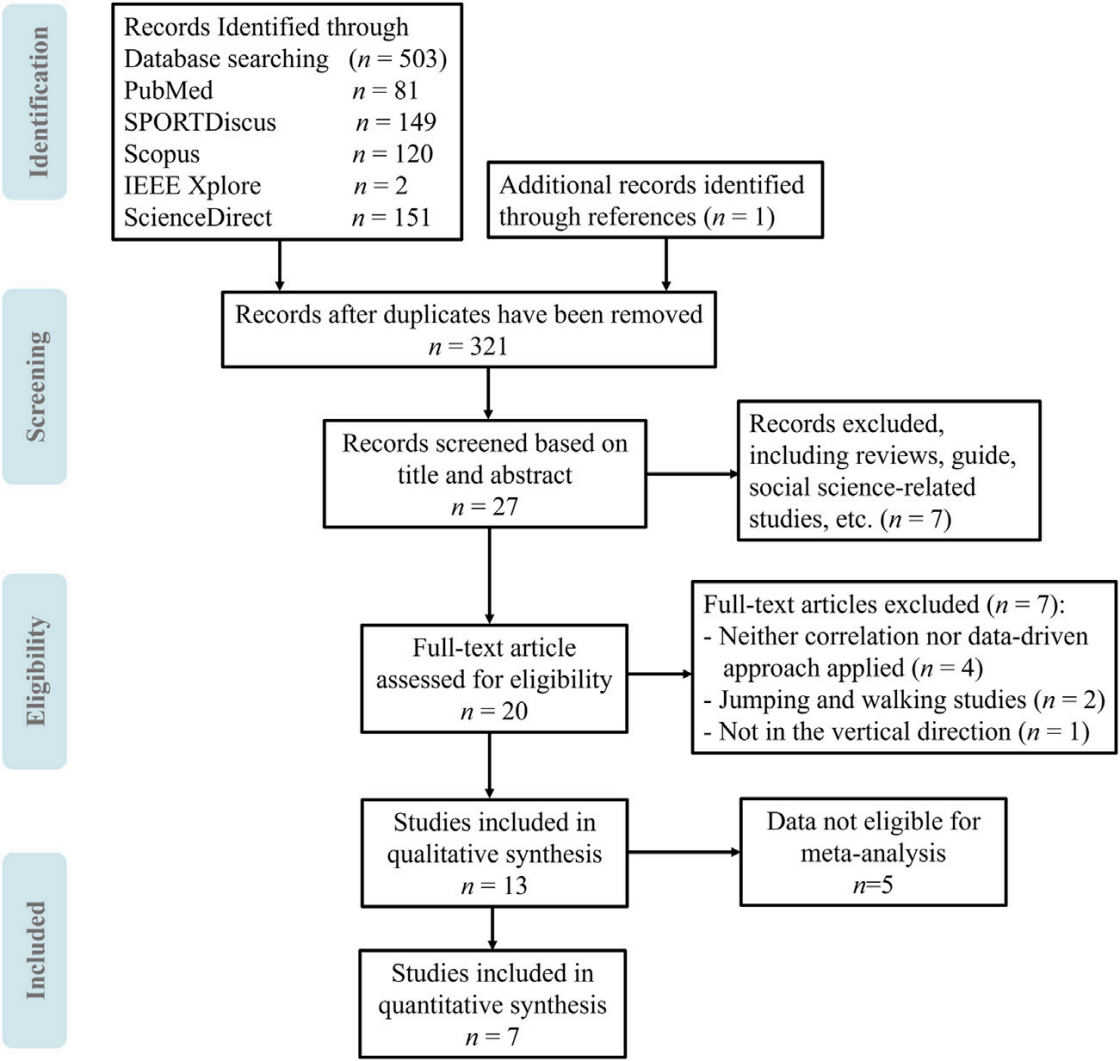
The Preferred Reporting Items for Systematic Reviews and Meta-Analysis (PRISMA) flow diagram illustrating the search strategy used in this review.

A total of 503 articles were identified via electronic databases retrieve (PubMed = 81, SPORTDiscus = 149, Scopus = 120, IEEE Xplore = 2, ScienceDirect = 151). Of these, 182 duplicate records were removed, and a further 294 articles were excluded based on the title and the abstract screening. Twenty-seven full-text articles were then evaluated, with seven being excluded. Reasons for exclusion included four articles not applying a correlation or data-driven approach, two focusing on jumping and walking studies, and one not addressing vertical direction. Five articles were not included in the quantitative synthesis due to data ineligibility for meta-analysis. The details of the search strategy are presented in Figure 2.

### 3.2 Quality assessment

The quality appraisal ratings for each article are presented in Table 2. Overall, the risk of bias was moderate. Methodological quality scores ranged from 14 to 17 out of a possible 18. The average quality assessment rate for the selected articles in this systematic review was 86.75%. The highest average quality assessment among the quality scores was 1.92 (Q2, Q4, and Q9), and the lowest was 1.38 (Q7). Additionally, seven articles were included in the meta-analysis (Laughton et al., 2003; Greenhalgh et al., 2012; Zhang et al., 2016; Cheung et al., 2019; Van den Berghe et al., 2019; Tenforde et al., 2020; Bradach et al., 2023).

**TABLE 2:** Quality assessment scoring of 13 included studies.

Study	Q1	Q2	Q3	Q4	Q5	Q6	Q7	Q8	Q9	Total	%	Mata
Tenforde et al. (2020)	+2	+2	+1	+2	+2	+2	+1	+2	+2	16	88.89	Yes
Cheung et al. (2019)	+2	+2	+2	+2	+1	+2	+2	+2	+2	17	94.44	Yes
Laughton et al. (2003)	+2	+2	+1	+2	+2	+1	+1	+2	+2	15	83.33	Yes
Van den Berghe et al. (2019)	+1	+2	+2	+2	+2	+2	+2	+2	+2	17	94.44	Yes
Zhang et al. (2016)	+1	+2	+1	+2	+1	+2	+2	+1	+2	14	77.78	Yes
Bradach et al. (2023)	+1	+2	+1	+2	+2	+1	+2	+2	+2	15	83.33	Yes
Greenhalgh et al. (2012)	+2	+1	+2	+2	+2	+2	+2	+2	+1	18	88.89	Yes
Matijevich et al. (2019)	+1	+2	+2	+2	+2	+1	+2	+2	+2	16	88.89	No
Zandbergen et al. (2023)	+2	+2	+1	+1	+2	+1	+1	+2	+2	14	77.78	No
Derie et al. (2020)	+2	+2	+2	+2	+2	+2	+2	+1	+2	17	94.44	No
Komaris et al. (2019)	+2	+2	+1	+2	+2	+2	+1	+2	+2	16	88.89	No
Tan et al. (2020)	+2	+2	+2	+2	+2	+1	N/A	+2	+2	15	83.33	No
Matijevich et al. (2020)	+1	+2	+2	+2	+2	+2	N/A	+2	+2	15	83.33	No
Average	1.62	1.92	1.54	1.92	1.85	1.62	1.38	1.85	1.92	15.62	86.75	\

*Note:* Mata = Inclusion in meta-analysis.

### 3.3 Study characteristics of data synthesis

As indicated in Table 3, seven studies included in this review assessed the relationship between TA and GRF metrics (Laughton et al., 2003; Greenhalgh et al., 2012; Zhang et al., 2016; Cheung et al., 2019; Van den Berghe et al., 2019; Tenforde et al., 2020; Bradach et al., 2023). Four studies (Zhang et al., 2016; Cheung et al., 2019; Tenforde et al., 2020; Bradach et al., 2023) were conducted on a treadmill, while three studies (Laughton et al., 2003; Greenhalgh et al., 2012; Van den Berghe et al., 2019) involved overground running. Two studies employed tri-axial accelerometers (Greenhalgh et al., 2012; Van den Berghe et al., 2019), one used a bi-axial accelerometer (Cheung et al., 2019), and one used a uniaxial accelerometer (Laughton et al., 2003), while two other studies utilized IMU sensors (Tenforde et al., 2020; Bradach et al., 2023). The frequency of IMU sensors was 1000 Hz in four studies (Greenhalgh et al., 2012; Cheung et al., 2019; Tenforde et al., 2020; Bradach et al., 2023), followed by 960 Hz in one (Laughton et al., 2003), 400 Hz in one (Zhang et al., 2016), and 100 Hz in another (Van den Berghe et al., 2019). Furthermore, the variable from IMU sensors was peak TA (in 7 studies), and the most common GRF variables were VILR (in 6 studies) (Greenhalgh et al., 2012; Zhang et al., 2016; Cheung et al., 2019; Van den Berghe et al., 2019; Tenforde et al., 2020; Bradach et al., 2023) and VALR (in 4 studies) (Laughton et al., 2003; Zhang et al., 2016; Cheung et al., 2019; Tenforde et al., 2020). Extremely strong (3 occurrences), strong (3 occurrences), medium (4 occurrences), weak (1 occurrence), and extremely weak (1 occurrence) correlations between peak TA and GRF metrics were reported in the seven collected literatures.

**TABLE 3:** Details of studies information of the relationship of tibial acceleration and GRF.

Study	Sample size (M/F) | age, height, mass	Running surface| speed | condition	Foot strike pattern	Sensor type and frequency (Hz)	Senor placement	Variables independent | dependent	Correlation coefficient
Tenforde et al. (2020)	169 (95/74) | age: 39 ± 13 years, height 1.72 ± 0.09 cm, mass: 70.4 ± 12.03 kg	Treadmill | 2.52 ± 0.25 m/s | Self-selected running shoes	FFS, MFS, and RFS	IMU sensor (IMeasureU), 1,000	The distal medial portion of the tibia above the medial malleolus	PTA, RPTA | VALR, VILR	PTA & VALR (r = 0.66–0.82), PTA & VILR (r = 0.66–0.73), RPTA & VALR (r = 0.47–0.67), RPTA & VILR (r = 0.37–0.67)
Cheung et al. (2019)	14 (7/7) | age: 26.4 ± 11.2 yrs, height 1.66 ± 0.09 cm, mass: 58.8 ± 9.7 kg	Treadmill | 2.78 m/s | Self-selected running shoes	RFS	Bi-axial accelerometer (ADXL278), 1,000	Anteromedial aspect of the tibia and aligned with the vertical axis of the tibia	PTA | VALR, VILR	PTA & VALR (r = 0.90), PTA & VILR (r = 0.91)
Laughton et al. (2003)	15 (NS) | age: 22.46 ± 4 years, height 1.79 ± 0.06 cm, mass: 66.41 ± 8.58 kg	Overground| 3.7 m/s ± 5%| Nike Air Pegasus	FFS and RFS	Uniaxial accelerometer (model 353B17), 960	Distal anteromedial aspect of the leg	PTA | VALR	FFS group (r = 0.70), RFS group (r = 0.47)
Van den Berghe et al. (2019)	13 (NS) | NS, height: 1.75 ± 0.08 m, mass: 70.6 ± 10.8 kg	Overground| 2.55, 3.20, and 5.10 ± 0.2 m/s | Li Ning Magne, ARHF041	RFS	MEMS tri-axial accelerometers (model LIS331), 100	Lower leg alongside the distal anteromedial aspect, 8 cm above the medial malleolus	PTA, RPTA | VILR	PTA & VILR (r = 0.64–0.84), RPTA & VILR (r = 0.57–0.61)
Zhang et al. (2016)	10 (8/2) | age: 23.6 ± 3.8 years, height: 1.73 ± 0.08 m, mass: 66.1 ± 12.7 kg	Treadmill (flat and ±10% inclination) | ± 15% of preferred speed | Adidas Adios Boost	NS	Accelerometers (Model 7523A5) 400	Anteromedial aspect of distal tibia	PTA | VALR, VILR	PTA & VALR (r = 0.49–0.91), PTA & VILR (r = 0.53–0.90)
Bradach et al. (2023)	28 (13/15) | age: 39 ± 13 years, height: 1.72 ± 0.09 m, mass: 68.5 ± 10.7 kg	Treadmill | Self-selected speed (2.81 ± 0.39 m/s) | Nike p-6000	NS	IMU sensor (IMeasureU, Blue Thunder), 1,000	Distal medial tibia, 1 cm above the medial malleolus	PTA | VILR	r = 0.31–0.80
Greenhalgh et al. (2012)	13 (10/3) | age: 30.0 ± 9.4 years, height 1.74 ± 0.06 m, mass: 70.6 ± 8.1 kg	Overground | 4 m/s ± 5% | Not mentioned	NS	Tri-axial accelerometer (Biometrics ACL300), 1,000	The distal anterior-medial aspect of the tibia and 8 cm above the medial-malleolus	PTA | VALR, VILR	PTA & VALR (r = 0.27), PTA & VILR (r = 0.47)

*Note:* FFS, forefoot strikers; MFS, midfoot strikers; RFS, rearfoot strikers; IMU, inertial measurement unit; PTA, peak tibial acceleration; RPTA, resultant peak tibial acceleration; VALR, vertical average load rates; VILR, vertical instantaneous load rates; NS, not specified; Extremely strong (0.8–1.0), strong correlation (0.6–0.8), medium correlation (0.4–0.6), weak correlation (0.2–0.4), extremely weak correlation (0–0.2).

### 3.4 Meta-analysis

#### 3.4.1 The correlation between overground and treadmill running

**FIGURE 3:**
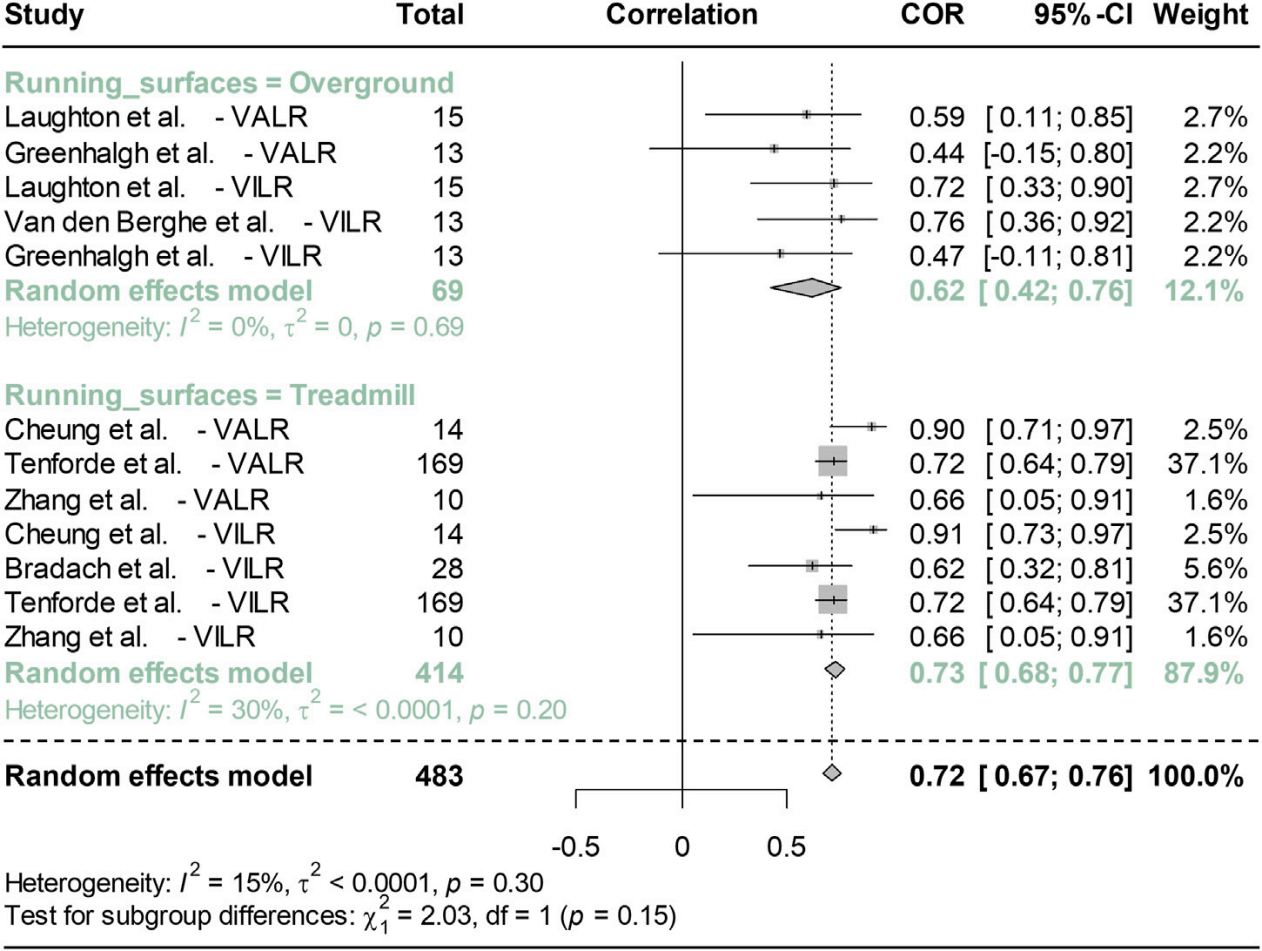
Meta-analysis compares the Pearson correlation coefficient between peak vertical acceleration and both VALR and VILR between overground and treadmill running. Note: VALR represents vertical average load rate, and VILR denotes for vertical instantaneous load rate.

Figure 3 presents a forest plot comparing the Pearson correlation coefficients between peak vertical TA and GRF, specifically VALR and VILR. The sensitivity analysis results were shown in Supplementary Material A (Supplementary Table SA1). For subgroup analysis, the moderating variable of running surfaces was considered, with the overground group comprising 3 studies (5 items) and the treadmill group consisting of 4 studies (7 items). In the overground and treadmill groups, the correlations were 0.62 and 0.73, respectively, with 95% confidence intervals (CI) of 0.42–0.76 for the overground group and 0.68 to 0.77 for the treadmill group. The I^2^ values were 0% for the overground group (*p* = 0.69) and 30% for the treadmill group (*p* = 0.3), indicating heterogeneity levels. The overall correlation between peak vertical acceleration and both VALR and VILR is 0.72, with a 95% CI of 0.67–0.76, and an I^2^ heterogeneity of 15% (*p* = 0.3).

#### 3.4.2 The correlation among different foot strike patterns

**FIGURE 4:**
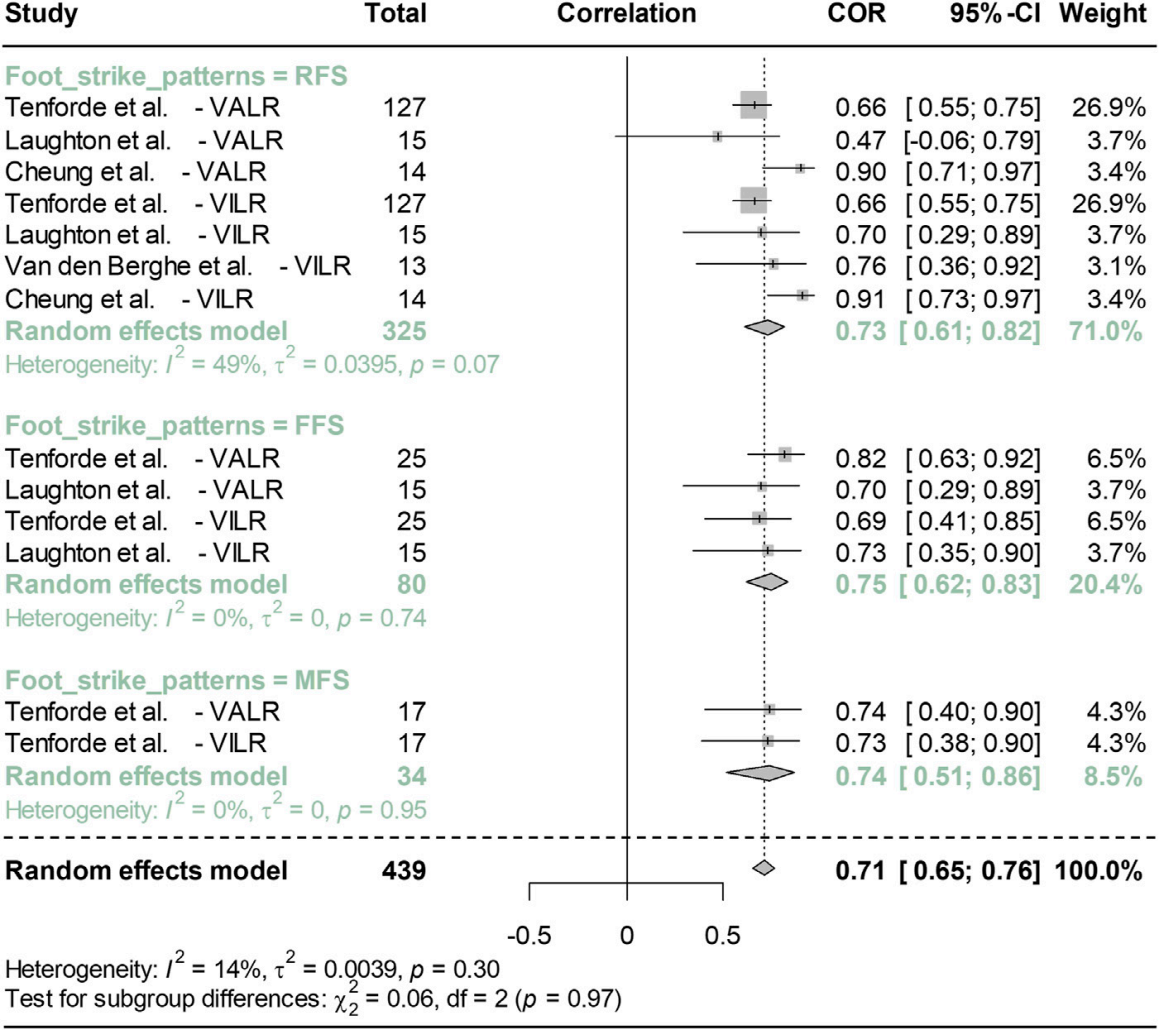
Meta-analysis compares the Pearson correlation coefficient between peak vertical acceleration and both VALR and VILR among different strike patterns. Note: VALR represents vertical average load rate, VILR denotes for vertical instantaneous load rate, RFS is rearfoot strike pattern, MFS is midfoot strike pattern, and FFS is forefoot strike pattern.

Figure 4 displays a forest plot comparing the Pearson correlation coefficients between peak vertical TA and both VALR and VILR across various foot strike patterns. The sensitivity analysis results were shown in Supplementary Materia1 A (Supplementary Table SA2). For the subgroup analysis, the foot strike pattern was used as a moderating variable. The RFS group included 4 studies (comprising 7 items), the FFS group encompassed 2 studies (4 items), and the MFS group consisted of 1 study (2 items). The correlations in the RFS, FFS, and MFS groups were 0.73, 0.75, and 0.74, respectively, with 95% confidence intervals (CI) of 0.61–0.82 for RFS, 0.62–0.83 for FFS, and 0.51–0.86 for MFS. The *I* ^*2*^ values indicated heterogeneity levels of 49% for the RFS group, and 0% for both the FFS and MFS groups. Collectively, the correlation coefficient across all groups was 0.71 with a 95% CI of 0.65–0.76, and an *I* ^*2*^ value of 14% (*p* = 0.3).

### 3.5 The relationship between TA/GRF, and tibial bone load

As shown in Table 4, two studies included in this review assessed the relationship between TA/GRF and tibial bone load (Matijevich et al., 2019; Zandbergen et al., 2023). Both studies were conducted on treadmills with participants wearing self-selected running shoes. Only one study reported the foot strike pattern as RFS (Zandbergen et al., 2023). In this study (Zandbergen et al., 2023), an IMU sensor, specifically the Xsens model with a sampling frequency of 240 Hz, was used to measure peak TA. Moreover, both studies utilized the Pearson correlation coefficient for correlation analysis. These studies explored correlations between GRF variables (weak correlations) and peak TA (extremely weak correlations) in relation to tibial load.

**TABLE 4:** Details of studies information of the relationship between tibial acceleration/GRF, and tibial bone load.

Study	Sample size (M/F) (kg)	Running surface | speed | condition	Foot strike pattern	Sensor type and frequency	Senor placement	Variables independent | dependent	Correlation coefficient
Matijevich et al. (2019)	10 (5/5) | age: 24 ± 2.5 years, height 1.7 ± 0.1 m, mass: 66.7 ± 6.4	Treadmill (level, uphill, and downhill) | 2.6–4.0 m/s | self-selected running shoes	NS	None	None	Impact peak, VALR | peak tibial force	Impact peak and peak tibial force (−0.29 ± 0.37); VALR & peak tibial force (−0.20 ± 0.35)
Zandbergen et al. (2023)	13 (8/4) | age: 36.7 ± 12.2 years, height 178.7 ± 9.6 cm, mass: 74.2 ± 17.7	Treadmill | 10, 12, and 14 km/h | self-selected running shoes	RFS	IMU sensor (Xsens), 240 Hz	Medial surface of the proximal tibia	PTA | maximum tibial compression force	0.04 ± 0.14

*Note:* GRF, ground reaction force; IMU, inertial measurement unit; PTA, peak tibial acceleration; VALR, vertical average load rates; RFS, rearfoot striker; NS, not specified; Extremely strong (0.8–1.0), strong correlation (0.6–0.8), medium correlation (0.4–0.6), weak correlation (0.2–0.4), extremely weak correlation (0–0.2).

### 3.6 Data-driving approaches

As presented in Table 5, three studies employed data-driven approaches to predict GRF metrics using acceleration data (Komaris et al., 2019; Derie et al., 2020; Tan et al., 2020), and one study used this approach to predict tibial loading force using IMU signals (Komaris et al., 2019). Additionally, three studies were conducted on treadmills (Komaris et al., 2019; Matijevich et al., 2020; Tan et al., 2020), and one was conducted overground (Derie et al., 2020). One study utilized IMU sensors (Tan et al., 2020), one used tri-axial accelerometers (Komaris et al., 2019), and two used virtual accelerometers (Derie et al., 2020; Matijevich et al., 2020), where the acceleration data were derived from kinematic measurements. Various data-driven methods were applied: gradient boosted regression trees (XGB) (Derie et al., 2020), artificial neural networks (ANN) (Komaris et al., 2019), convolutional neural networks (CNN) (Tan et al., 2020), and LASSO regression (Matijevich et al., 2020). The studies consistently showed high predictive accuracy: mean absolute percentage error (MAPE) was below 10% in two studies (Derie et al., 2020; Matijevich et al., 2020), normalized root mean square error (NRMSE) was under 10% in one study (Tan et al., 2020), and RMSE remained less than 0.2 BW across all (Komaris et al., 2019).

**TABLE 5:** Details of studies information of data-driving approaches.

Study	Sample size (M/F) (kg)	Running surface | speed | condition	Foot strike pattern	Sensor type and frequency	Senor placement	Variables predictor | response	Machine learning algorithm	Accuracy
Derie et al. (2020)	93 (55/38) | age: 35.3 ± 10.0 years, height: 1.73 ± 0.07 m, mass: 68.6 ± 8.8	Overground | 2.55 m/s, 3.20 m/s and 5.10 m/s | Li Ning Magne, ARHF041	NS	Tri-axial accelerometers (LIS331), 1,000 Hz	Antero-medial side of the tibia	PTA | VILR	XGB	MAPE: 6.08%
Komaris et al. (2019)	28 (27/1) | age: 34.8 ± 6.6 years, height: 176 ± 6.7 cm, mass: 69.6 ± 7.6	Treadmill | 2.5, 3.5 and 4.5 m/s | Not mentioned	NS	Virtual accelerometer (deriving acceleration from kinematics)	Shank	Tri-axial tibial acceleration | vertical GRF, anteroposterior GRF, mediolateral GRF	ANN	RMSE: vertical GRF = 0.13 B W, anteroposterior GRF = 0.04 B W, and mediolateral GRF = 0.04 B W
Tan et al. (2020)	15 (8/7) | age: 23.9 ± 1.1 years, height: 1.68 ± 0.08 m, mass: 61.9 ± 7.7	Treadmill | 2.4 and 2.8 m/s | standard and minimalist running shoes	FFS, MFS, and RFS	IMU sensor (Xsens), 200 Hz	One-third of the distance between keen and ankle joints	Tri-axial linear acceleration and angular velocity | VALR	CNN	NRMSE = 9.7 ± 3.6%
Matijevich et al. (2020)	10 (5/5) | age: 24 ± 2.5 years, height: 1.70 ± 0.1 m, mass: 67 ± 6	Treadmill (±9 inclination) | 2.6–4.0 m/s | self-selected shoes	NS	Virtual accelerometer (deriving acceleration from kinematics)	Shank	Sagittal joint angle at midstance | peak tibial force	LASSO regression	MAPE = 8.0 ± 2.9%

*Note:* LASSO, least absolute shrinkage and selection operator; XGB, gradient boosted regression trees; ANN, artificial neural network; CNN, convolutional neural networks; MAPE, mean absolute percent error; NRMSE: normalized root mean square error; MAE, mean absolute error; Adam = adaptive moment estimation; IMU, inertial measurement unit; PTA, peak tibial acceleration; VILR, vertical instantaneous loading rate; FFS, forefoot strikers; MFS, midfoot strikers; RFS, rearfoot striker; NS, not specified.

## 4 Discussion

This review critically evaluates the correlation between tibial acceleration, ground reaction forces, and tibial bone loading in running. It highlights the mixed results obtained from existing research in this domain and emphasizes the marginal link found between these biomechanical factors and tibial bone stress. The discussion also underscores the pivotal role of wearable sensor technology in measuring these forces, and its potential when combined with machine learning techniques, in redefining our approach to monitoring, preventing, and rehabilitating running-related injuries.

### 4.1 Peak tibial acceleration and impact loading rate

The body segment acceleration is shaped by GRF and dampening from bodily shock absorbers. Capturing peak positive acceleration at distal locations allows measurement before attenuation as the shock wave propagates proximally. Notably, vertical acceleration correlates directly with vertical GRF: higher vertical GRF load rate leads to increased vertical axial acceleration prior to attenuation (Lafortune et al., 1995). This findings from the data synthesis analysis showed only moderate correlation of coefficient between peak TA and GRF loading rate, which does not support with the general hypothesis under many studies that peak TA is an indicator of impact loading rate (Bigelow et al., 2013; Lucas-Cuevas et al., 2017; Raper et al., 2018; Cheung et al., 2019; Van den Berghe et al., 2019; Johnson et al., 2021; Ryu et al., 2021; Bradach et al., 2023; Johnson et al., 2023; van Middelaar et al., 2023; Zandbergen et al., 2023). This aligns with findings from the meta-analysis in this study, particularly for overground running.

The prevailing hypothesis in gait retraining research posits a robust positive correlation between the vertical GRF load rate and TA (Cheung et al., 2019; Tirosh et al., 2019; Sheerin et al., 2020; Van den Berghe et al., 2020; Derie et al., 2022). This assumption underpins studies suggesting that mitigating peak TA could be instrumental in reducing overuse injury risks by concurrently diminishing the load rate (Milner et al., 2006; Huang et al., 2019; Tavares et al., 2020; Warden et al., 2021). However, reliance on this correlation as a foundation for gait retraining strategies may result in oversimplified approaches that overlook the complexities of individual gait patterns and the multifaceted nature of injury risk factors (Pohl et al., 2008; van Gelder et al., 2023).

### 4.2 The correlation between GRF or acceleration and tibial bone load

TA is often used as a proxy for impact forces during running because it's relatively easy to measure, especially with the advent of wearable technology (Ryu et al., 2021; Xiang et al., 2022c; Xiang et al., 2022d; Bradach et al., 2023; van Middelaar et al., 2023; Xiang et al., 2023). However, the relationship between external forces (such as GRF and TA) and internal stresses (such as bone loading) is not always straightforward (Matijevich et al., 2020). Several factors can influence this relationship. Individual biomechanics, such as gait patterns, muscle strength, and joint stability, can significantly alter how external forces are translated into internal stresses (Baggaley et al., 2022). Moreover, the body’s adaptive responses to running, such as increased bone density or changes in soft tissue properties, can also affect this relationship. These adaptations can provide a buffering effect, reducing the impact of external forces on internal structures. A more holistic approach that considers both external forces and individual biomechanical factors could be more effective in understanding and preventing running-related injuries.

Concerning the relationship between GRF and internal bone loads, it is pertinent to note that recent studies, including those by Zandbergen et al. (2023); Matijevich et al. (2019), have provided compelling evidence challenging the traditionally assumed strong correlation. Zandbergen et al. (2023) found no significant correlation between acceleration and internal bone loads in the tibia, nor between GRF features and tibial bone load during running. Consistent with these findings, our meta-analysis demonstrates that peak TA does not directly correlate with the external loading rate. Further, Matijevich et al. (2019) substantiated that GRF metrics are not consistently correlated with tibial bone load across varied running speeds and slopes, thereby questioning the reliability of GRF as a predictor of internal bone stress in different running conditions. Considering that tibial compression forces encompass both external and internal forces, internal biomechanical adaptations may impact internal forces, even in the presence of external overload, thus influencing the prevention of related injuries (Baggaley et al., 2022). This is supported by recent studies (Milner et al., 2006; Van der Worp et al., 2016; Milner et al., 2023). These insights necessitate a reconsideration of existing biomechanical models and wearable technology applications in running injury prevention. It also highlights that the strategy of reducing peak TA or GRF to mitigate tibial stress fracture risk may be misleading (Van der Worp et al., 2016; Zandbergen et al., 2023).

In the realm of running biomechanics, the interplay between neuromotor control and muscle co-contraction presents a critical avenue for understanding the complex dynamics of tibial acceleration, GRF, and tibial bone loading. The coordinated muscle actions, steered by sophisticated neuromotor control, significantly dictate the force distribution and magnitudes transmitted through the musculoskeletal system during running (Kellis et al., 2011; Di Nardo et al., 2015). Insights from Martelli et al. (2011) shed light on how sub-optimal neuromotor strategies can amplify joint loads, potentially leading to increased tibial bone stress in runners. Furthermore, while muscle co-contraction is crucial for joint stabilization, it's important to note that excessive co-contraction might paradoxically decrease stability by increasing the mechanical loads on the tibia, without proportionally enhancing stability (Benjuya et al., 2004; Cenciarini et al., 2009; Tassani et al., 2019). This highlights the importance of identifying an optimal level of muscle co-contraction that ensures joint stability without contributing to unnecessary stress, aligning with the perspectives offered by Martelli et al. (2011).

The advent of wearable sensor technology, capable of capturing these complex neuromotor and muscle dynamics in real-time, opens up new vistas. By amalgamating this data with traditional measures such as GRF and TA, wearable sensors can offer a more nuanced understanding of running biomechanics. This comprehensive approach not only challenges traditional paradigms but also heralds a new era of integrated strategies in monitoring, preventing, and rehabilitating running-related injuries, emphasizing the shift towards more holistic models in running biomechanics studies.

### 4.3 Data-driven approach to external and internal predictions

The ongoing progression in machine learning and wearable technology has facilitated the innovative use of data from inertial sensors, particularly in the prediction of GRF metrics (Higgins et al., 2003; Cheung et al., 2019; Hernandez et al., 2021). This advancement is notable in its potential to offer a more dependable methodology compared to approaches reliant on the correlation between peak TA and impact loading rate. The latter method’s assumption of a strong correlation may not always hold true (Laughton et al., 2003; Greenhalgh et al., 2012; Zhang et al., 2016), underscoring the significance of this novel application of inertial sensor data in biomechanics studies.

Nevertheless, caution is warranted when asserting that reducing the impact loading rate could effectively mitigate musculoskeletal injuries in running, such as tibial stress fractures (Milner et al., 2006; Milner et al., 2007; Matijevich et al., 2019; Milner et al., 2023). The data-driven approach has also yielded favorable outcomes in projecting tibial bone force using wearable sensor data (Matijevich et al., 2020; Elstub et al., 2022). This approach incorporates the muscular forces acting on the tibia, potentially offering a more comprehensive understanding of musculoskeletal injuries (Matijevich et al., 2019). By integrating this data with external impact loading rates, a more holistic view of the biomechanical factors contributing to injury risk can be achieved, enhancing the precision and effectiveness of injury prediction and prevention strategies. Although data-driven approaches using wearable sensors show promise for predicting external loading (Derie et al., 2020; Tan et al., 2020) and internal muscular force (Matijevich et al., 2019; Matijevich et al., 2020), their opaque “black-box” nature presents a challenge in terms of data interpretability or explainable artificial intelligence (XAI) (Halilaj et al., 2018; Uhlrich et al., 2023). This area warrants further investigation to understand how wearable sensor signals correlate with biomechanical forces (Kaji et al., 2019; Schlegel et al., 2019; Jeyakumar et al., 2020; Gandin et al., 2021; Xiang et al., 2024). Therefore, personalized biomechanical adaptation strategies, tailored for precise injury prevention and rehabilitation monitoring, can be more effectively applied once a deeper understanding of these correlations is achieved.

### 4.4 Implications for future studies

➢ The utility of peak TA as an indicator of GRF, particularly VALR and VILR during running, is subject to skepticism in the context of current literature, especially with respect to overground running.
➢ A moderate to strong correlation exists between peak TA and vertical loading rate, irrespective of the foot strike patterns. However, it is important to note that the sample sizes for RFS and MFS are relatively limited, warranting caution in generalization of these findings.
➢ Strategies for gait retraining that focus on diminishing loading rates through the reduction of peak TA may not be adequately supported by empirical evidence.
➢ While a correlation between peak TA and impact loading is observed, this does not necessarily imply a direct linear relationship between either GRF or TA and the internal forces exerted on the tibial bone.
➢ Data-driven models, which utilize acceleration data from inertial wearable sensors, exhibit a proficient capability in accurately predicting both external impact loading and internal tibial bone loading.
➢ Future studies should focus on enhancing XAI to augment interpretability of data-driven biomechanical models. This advancement is crucial for effectively correlating wearable sensor data with biomechanical forces.
➢ Embracing multifactorial methodologies that integrate insights from biomechanics, data science, kinesiology, and clinical practice not only minimizes confounding factors but also enriches the interpretation and applicability of research outcomes in real-world settings.

## 5 Conclusion

In conclusion, this study critically assesses the relationship between TA, GRF, and tibial bone loading in the context of running. It highlights the limited correlation between these biomechanical factors and tibial bone stress, challenging traditional beliefs. The research underscores the significant potential of wearable sensors and machine learning in advancing our understanding of running biomechanics. These technologies offer promising avenues for injury monitoring, prevention, and rehabilitation, suggesting a need for a shift towards more integrated and holistic approaches in running biomechanics.
