# Supplementary material for: Rethinking running biomechanics: a critical review of ground reaction forces, tibial bone loading, and the role of wearable sensors
Source: Front Bioeng Biotechnol. 2024 Apr 8;12:1377383. doi: 10.3389/fbioe.2024.1377383 (PMC11033368; doi:10.3389/fbioe.2024.1377383)
Supplement: Supplementary file 1 [file Table1.docx]

Supplementary Material A

# Supplementary Tables

**Table SA1.** Sensitivity analysis results of the Pearson correlation coefficient between peak vertical acceleration and loading rate between overground and treadmill running through sequential exclusion of studies by weight.

| Running surface | Removed study | Weight in total | Correlation of coefficient | 95% CI | *I^2^* |
| --- | --- | --- | --- | --- | --- |
| overground |  | 12.1% | 0.62 | [0.42, 0.76] | 0% |
|  | Van den Berghe et al. 2019 | 9.9% | 0.58 | [0.34, 0.74] | 0% |
|  | Greenhalgh et al.  2012 | 5.5% | 0.66 | [0.37, 0.83] | 0% |
| Treadmill |  | 87.9% | 0.73 | [0.68, 0.77] | 30% |
|  | Zhang et al.  2016 | 84.7% | 0.73 | [0.68, 0.77] | 52% |
|  | Cheung et al.  2019 | 79.7% | 0.71 | [0.66, 0.76] | 0% |
|  | Bradach et al.  2023 | 74.1% | 0.72 | [0.66, 0.77] | 0% |

**Table SA2.** Sensitivity analysis results of the Pearson correlation coefficient between peak vertical acceleration and loading rate among different strike patterns running through sequential exclusion of studies by weight.

| Running surface | Removed study | Weight | Correlation of coefficient | 95% CI | *I^2^* |
| --- | --- | --- | --- | --- | --- |
| RFS |  | 71.0% | 0.73 | [0.61, 0.82] | 49% |
|  | Van den Berghe et al.  2019 | 67.9% | 0.73 | [0.59, 0.83] | 56% |
|  | Cheung et al.  2019 | 61.7% | 0.65 | [0.58, 0.72] | 73.9% |
|  | Laughton et al.  2003 | 53.7% | 0.66 | [0.58, 0.72] | 0% |
| MFS |  | 20.4% | 0.75 | [0.62, 0.83] | 0% |
|  | Laughton et al.  2003 | 13.0% | 0.76 | [0.60, 0.86] | 5% |
| FFS |  | 8.5% | 0.74 | [0.51, 0.86] | 0% |

Note: RFS: rearfoot strike pattern, MFS: midfoot strike pattern, and FFS: forefoot strike pattern.
